# Hand Motion Control Ability Between Young and Older Adults: Comparative Study

**DOI:** 10.2196/65224

**Published:** 2025-07-21

**Authors:** Jung-Soon Kim, Hui-Jun Kim, Minju Kim, Sung-Hee Kim

**Affiliations:** 1 Department of Artificial Intelligence Dong-eui University Busan Republic of Korea; 2 Department of IT Convergence Dong-Eui University Busan Republic of Korea; 3 Department of Industrial ICT Engineering Dong-Eui University Busan Republic of Korea

**Keywords:** youth vs elders, hand gesture, hand motion control, older adults, young adults, hand movements

## Abstract

**Background:**

Age-related differences in motor skills have been extensively studied, with growing interest in using behavioral data for cognitive assessment. Compared to traditional tools like the Mini-Mental State Examination or Cognitive Impairment Screening Test, behavior-based methods offer the advantage of shorter testing durations, less learning effects, and continuous data tracking. Hand movements, in particular, provide a practical way to gather motor performance data with fewer spatial constraints. This study aims to explore whether hand rotation movement can effectively distinguish age-related motor skill differences, with future applications potentially extending to cognitive assessments, including early detection of mild cognitive impairment.

**Objective:**

This study investigates whether hand rotation movements can be used to distinguish 2 age groups, young adults (aged 20-29 years) and older adults (aged 65-80 years). We hypothesize that differences in hand motion control ability will exist between the 2 groups. In total, 7 hand motion measurement indicators related to single hand test indicators, time comparison indicators between rotations, and angle comparison indicators between rotations were defined to test this hypothesis, aiming to identify meaningful indicators for older adults experiencing normal aging before conducting experiments on patients with mild cognitive impairment or dementia.

**Methods:**

A total of 68 participants, 39 older adults (aged 65-80 years) and 29 young adults (aged 20-29 years), all capable of normal arm, hand, and finger movements, participated in the experiment. Participants sat facing a webcam and were asked to perform hand rotation movements as quickly and accurately as possible with both hands for 10 seconds. They performed 3 trials with a 30-second break in between. For statistical verification, we set the significance level at .05 and analyzed the data using the generalized estimation equations model to assess the effects of the between-subject factor (age group: younger vs older) and the within-subject factors (hand: left vs right, and trials 1, 2, and 3).

**Results:**

Among the 7 measured indicators, 3 (total rotation count, angle, and time) showed statistically significant differences between age groups. Younger participants performed more rotations (B=5.29, *P*=.002), demonstrated a greater range of motion (B=1334.37, *P*=.007), and completed the task in less time (B=0.99, *P*=.003), indicating age-related differences in upper limb motor function. Trial order also had a significant main effect on rotation count and angle. Trial 1 differed significantly from trials 2 and 3, while no difference was observed between trials 2 and 3, suggesting that trial 1 may reflect a practice effect.

**Conclusions:**

The findings revealed that the older adult group demonstrated statistically significant differences compared to the young adult group in their ability to control hand rotation movements. A learning effect was observed across the 3 trials, suggesting that the first trial should be discarded for use as a stable measurement.

## Introduction

### Background

Recently, a growing number of studies have focused on digital biomarkers for the early detection and intervention in preclinical stages of mild cognitive impairment (MCI) and dementia due to their advantages, such as low cost, time efficiency, and high accessibility [1,2]. Traditional detection methods include imaging assessments (eg, positron emission tomography) and biological evaluations (eg, cerebrospinal fluid analysis), but these methods have limitations such as being invasive, inconvenient, time-consuming, expensive, and difficult to access [3]. Furthermore, while clinical and neuropsychological assessments remain key standards, they also have limitations, including long testing intervals to prevent learning effects [4], reliance on self-reporting, and interevaluator variability [5]. In contrast, digital biomarkers are defined as objective and quantifiable physiological and behavioral data collected through digital devices, enabling frequent or continuous evaluation with minimal disruption to daily life. This allows for objective, ecologically valid, and long-term follow-up in preclinical detection processes [6-9]. Developing new digital biomarkers that fulfill these requirements is important.

Research on digital biomarkers based on behavioral data has primarily focused on gait or hand movement data [10]. In particular, hand movement-based studies are gaining attention due to their relative advantages compared to gait, such as requiring less space [11,12], ensuring physical safety for participants (ie, gait poses a risk of falls), and using low-cost data measurement devices. However, previous studies have either measured imitation accuracy of predefined static hand movements using artificial intelligence (AI) [13] or collected dynamic hand movement data similar to daily activities using virtual reality (VR) devices [14,15]. These methods face limitations in overcoming the drawbacks of traditional biomarkers. Challenges include the occurrence of learning effects from repetitive presentation of fixed movements in repeated measurement processes, the high cost of VR devices, and psychological barriers stemming from the use of unfamiliar equipment. As a result, these methods lack sufficient validity verification required for their application as digital biomarkers in cognitive ability assessments.

To address these limitations, we used a hand rotation-based digital biomarker inspired by the correlation between upper limb motor skill capability and cognitive function. Numerous prior studies have demonstrated that cognitive impairment leads to a decline in upper limb motor abilities [16-19]. The hand rotation-based movement reflects hand motion control ability that has minimal learning effects [13] and can be measured by images with an AI-based measurement system to collect quantitative data, thereby minimizing interevaluator variability [20]. To quantitatively measure hand rotation data, several measures can be used, such as total rotation count, total rotation time, and symmetry [21], as well as derived indicators, such as changes in amplitude and variations in performance time. Before conducting experiments on patients with MCI or dementia, we investigated if the hand rotation-based digital biomarker could identify different age groups, based on the understanding that healthy aging induces neurochemical and structural changes in older adults [22], which consequently impacts their motor performance, leading to reduced motor control and coordination [23,24].

### Objective

To achieve this, our objective is to validate the proposed hand rotation-based digital biomarker to distinguish differences between a young adult group and an older adult group. We used a mixed design to investigate the effects of age, hand dominance, and repeated trials on motor performance. The significance of this research lies in overcoming the economic and spatial limitations of existing behavior-based digital biomarkers and developing a more accessible digital biomarker. Additionally, this study provides direction and insights for future digital biomarker development.

## Methods

### Ethical Considerations

#### Ethical Approval and Participant Recruitment

The study was conducted with the approval of the Dong-eui University institutional review board (DIRB-202307-HR-E-20) to respect and protect the safety, well-being, and rights of the participants.

The selection of study participants was based on a similar study on hand motion measurement [25]. In this experiment, participants were divided into experimental and control groups, and the experiment involved repeatedly tapping finger-to-finger movements. This study evaluates hand movement control abilities, with the experimental group being older adults aged 65-80 years and the control group being young adults aged 20-29 years. Older adult participants were selected based on their cognitive status, ensuring they were not diagnosed with MCI or dementia and could perform daily arm, hand, and finger movements. A 2-sided test was conducted with a significance level of .05 and a power of over 70%. To achieve similar significant results, 31 young adults and 39 older adults were selected for evaluation in each group. Older adult participants were recruited through a public announcement at the Dasarang Cultural Complex Arts Center in Busan, which is a community welfare center operated by the local government. The young adult participants were selected from among the undergraduates at Dong-eui University through a flyer.

#### Informed Consent and Privacy Protection

Participants were fully informed about the purpose, procedures, and any potential risks or discomforts associated with the study. They were informed that video would be recorded and the data would be analyzed and used only for this study. They were also informed that they could withdraw from the study at any time without any disadvantage. Written consent was obtained only from those who voluntarily agreed to participate. Participants’ names were replaced with anonymized identifiers, and only minimal clinical information, such as age, gender, and physical characteristics, was collected.

The collected information and data were securely stored on a personal portable storage device (USB) and were immediately destroyed if a participant withdrew or upon completion of data analysis. The data were not used for any other purpose.

#### Rewards

The compensation for the experiment was a dietary supplement worth 20,000 KRW (US $14.72) per person. Additionally, participants were informed that they would receive the compensation even if they did not meet the normal range criteria for the Purdue Pegboard Test (PPT) or if they had to stop in the middle and could not complete the study.

### Study Design

This study used a mixed-design method to investigate the effects of age, hand dominance, and repeated trials on motor performance. The experiment included both between-subject and within-subject factors. The between-subject factor was age group, with participants divided into 2 groups: younger adults (ages 20-29 years) and older adults (ages 65-80 years). This design allows for the examination of age-related differences in motor function. The within-subject factors included hand (right vs left) and trial (3 repeated trials). Each participant completed the task using both hands. The task was repeated 3 times (trial 1, trial 2, and trial 3) to assess potential changes in performance due to repetition, learning, or fatigue. Participants were asked to perform a hand rotation task, in which they rotated their hands for a total of 10 seconds. The outcome variables measured are explained in Table 1. These measures were collected across both hands and trials for each participant, providing a comprehensive assessment of motor function. By using a mixed design, this study allowed for the analysis of both between-subject effects (comparing younger and older participants) and within-subject effects (comparing performance across hands and trials), as well as their interactions. The design also enabled us to investigate whether performance improvements across trials, indicative of a learning effect, differed between the age groups.

**Table 1:** Measurements.

No	Measurements	Explain
1	Total rotation count	Total number of hand rotations in 10 seconds^a^
2	Total rotation time	Cumulative total of each rotation time^a^
3	Total rotation time change^b^	Sum of the amount of time change that occurs while rotating the hand
4	The number of rotation time changes	The number of times the time changes as the hand rotates^a^
5	Total rotation angle	Cumulative total of on rotation time
6	Total rotation angle change^c^	The sum of the angular changes that occur while rotating the hand^a^
7	The number of rotation angle changes	The number of times the angle changes as you rotate your hand^a^

^a^Rotation unit = 1 rotation.

^b^TCA: time of rotation change amount.

^c^ACA: angle of rotation change amount.

### Statistical Analysis

#### Model Specification

Generalized estimating equations (GEE) were used to estimate causal models for panel data or across entire panels, particularly for multivariate variables that deviate from a normal distribution by applying the generalized linear model. GEE is a technique capable of handling repeated measures and time-series data, which are challenging for generalized linear models [26]. All statistical analyses were conducted using Python (version 3.10) on Google Colab (Ubuntu; version 22.04) with the following libraries: pandas (version 2.2.2; developed by the pandas community, under the NumFOCUS umbrella) for data manipulation, statsmodels (version 0.14.4; developed by the statsmodels community of contributors) for statistical modeling to fit GEE models, scipy (version 1.13.1; developed by the SciPy community, under the NumFOCUS umbrella) for additional statistical functions, and pingouin (version 0.5; developed by the Pingouin project team) for post hoc pairwise comparisons between trials to examine potential learning effects. By leveraging GEE, we effectively controlled for within-subject correlations inherent in repeated measures designs, and post hoc pairwise comparisons were performed to identify significant differences across trials. The application of Bonferroni adjustments ensured that the results remained robust against type I error due to multiple comparisons.

#### Data Exclusion

Before the main experiment, the PPT [27] was conducted to verify that there were no abnormalities in the arm, hand, and finger movements of the participants. Participants falling out of the range of the benchmark in Table 2 were excluded from the hand movement control ability experiment. To note, while recruiting, we informed participants that they should not have been diagnosed with MCI. However, we did not administer an additional test, and participation was voluntary.

**Table 2:** Purdue Pegboard Test average scores by age group (n=158).

Age	Male, mean (SD)	Female, mean (SD)
20-29 years old	12.8 (2.9)	13.6 (0.9)
60 years and older	7.9 (1.7)	8.2 (1.8)

### Overview of Hand Movement Measurement System

#### Defining Hand Rotation Behavior

**Figure 1:**
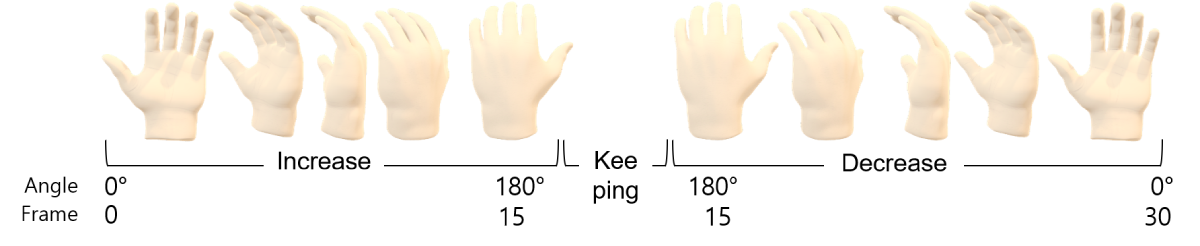
Example of hand rotation for 360° over 30 frames. Hand rotation is defined from 0° (palm facing forward) to 180° (back of hand facing forward). Hand rotation consists of 3 phases: increasing, maintaining, and decreasing. One full rotation includes 180° in each direction (total 360°), measured over 30 frames (~0.03 s per frame).

Hand rotation is defined as having a rotation angle of 0° when the palm is facing forward toward the webcam system and a rotation angle of 180° when the back of the hand is facing forward. Hand rotation consists of 3 stages based on the change in hand rotation angle: increase, maintain, and decrease. One full rotation is defined as rotating 180° in one direction and then 180° in the opposite direction (Figure 1). The total rotation angle is 360°, and the video consists of 30 frames, with each frame lasting approximately 0.03 seconds.

**Figure 2:**
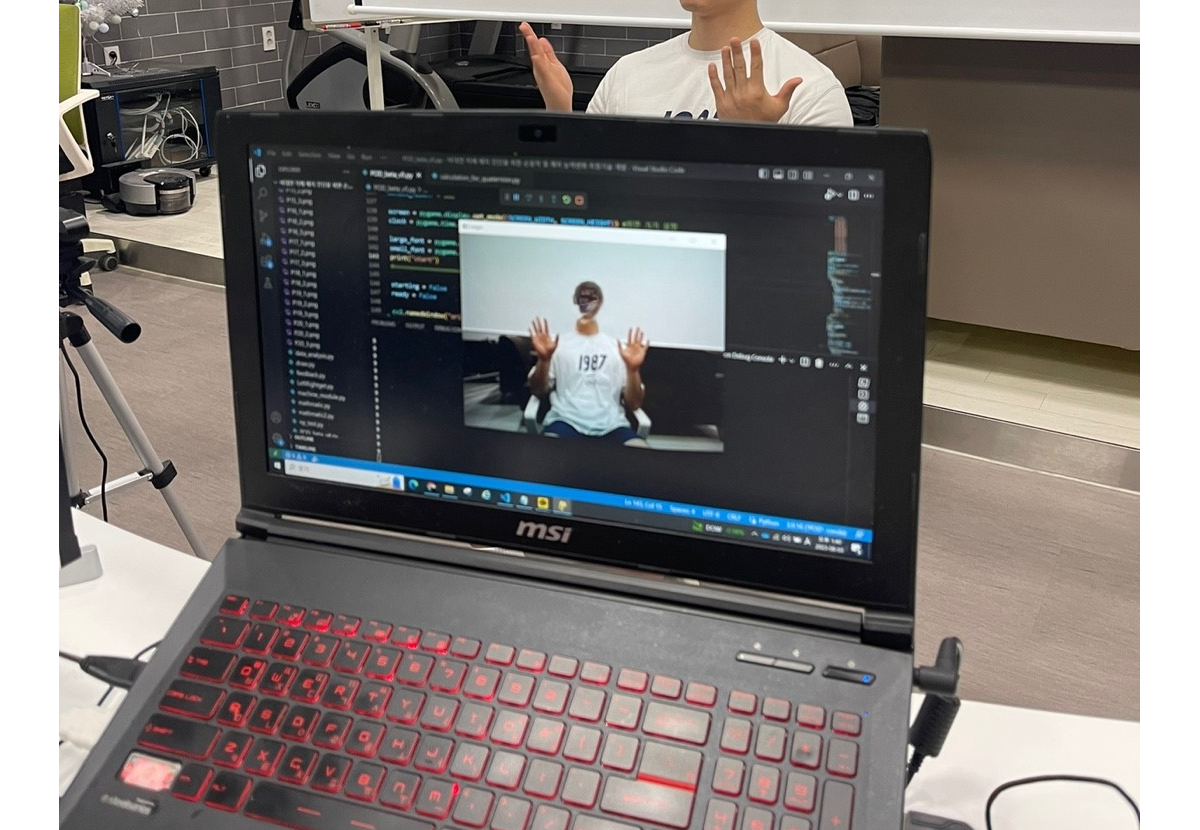
Experiment setting. This figure shows the experimental setup for the comparative study on hand movement control abilities between younger and older adults. In the experiment, participants sat facing the webcam, and upon the appearance of a "recording" sign on the screen, they were instructed to perform hand rotation movements with both hands as quickly and accurately as possible for 10 seconds. This procedure was repeated 3 times. The experiment for the older adult group was conducted at a welfare facility operated by the local government (Dasarang Cultural Complex Arts Center in Busan), while the experiment for the younger adult group was conducted with university students enrolled at Dong-eui University.

#### Measurement System

**Figure 3:**
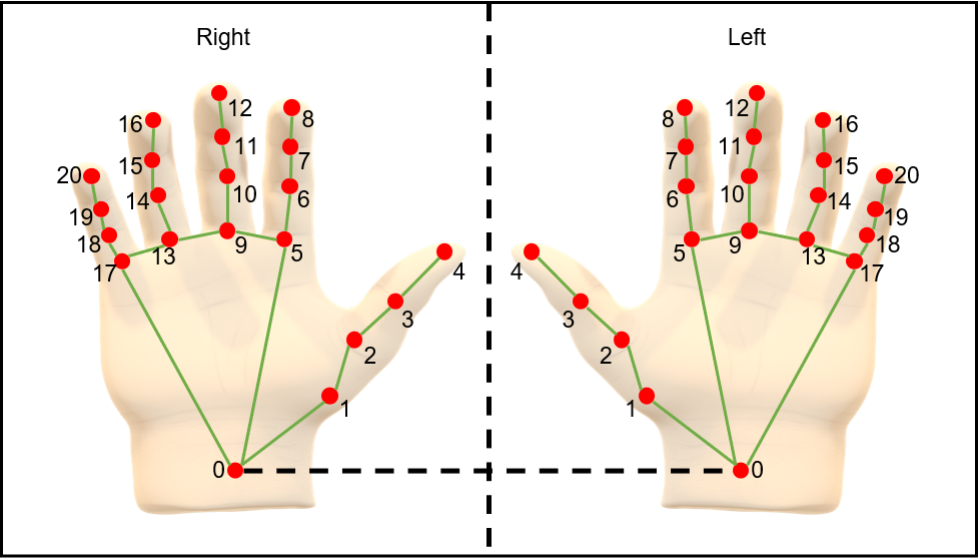
Landmarks based on hand shape. Visualization of the 21 hand landmarks.

The main experiment used an AI-based video processing hand rotation measurement technology [20]. This system involves the participant sitting in front of a webcam and performing hand rotation movements in real time for 10 seconds (Figure 2). The video data were recorded, and the system recognized the hand skeleton to collect and analyze the data. Using real-time video information captured by the webcam, the system estimated the 3D coordinates (x, y, z) of 21 hand joints (landmarks), as shown in Figure 3, using the MediaPipe Hands model Application Programming Interface [28]. The extracted coordinates were used to calculate the position vector through vector operations between the tip of the thumb (landmark 4) and the wrist (landmark 0). The rotation angle of the hand is determined by using the change in the position vector's movement along the Z-axis over time (frames). The algorithm for calculating the hand's rotation angle involves inverse calculating the quaternion [29]. To measure 1 full rotation of the hand, the reversal of the movement direction of the aforementioned position vector is used. Since the sign (negative or positive) of the quaternion’s slope (5 frames) is the same as the vector movement direction, 1 full rotation of the hand can be measured according to the sign reversal of the slope value. Ultimately, the landmark coordinates of the fingers, the video file captured by the webcam, and the 7 measurements explained in Table 1 (ie, total rotation count, total rotation time, total rotation time change, the number of rotation time changes, total rotation angle, total rotation angle change, and the number of rotation angle changes) were automatically saved to the computer.

#### Experimental Procedure

This study conducted a comparative experiment on hand motion control ability between young adults and older adults. Prior to the main experiment, (1) a recruitment notice was posted to recruit study participants. On the day of the experiment, when the experimenter visited the experiment site, (2) the experimenter thoroughly explained the experiment to the participants using an explanation document and received a consent form, and (3) written consent for the provision of personal information was obtained from the participants. Then, (4) for the PPT (preliminary experiment), participants sat at a desk in a comfortable chair facing the researcher. Older adult participants were encouraged to use magnifying glasses if they had difficulty seeing the holes in the pegboard. Before the main experiment, participants practiced inserting 3-4 pins into the pegboard to correct any mistakes, and then the 30-second test was conducted. The experiment began when the researcher said “start” and ended after 30 seconds, with the number of pins inserted being recorded. This process is repeated 3 times. (5) For the hand motion control measurement action (main experiment), the researcher sat in front of a laptop, with the webcam facing the participant. The participant sat in a chair facing the webcam. They were instructed to perform hand rotation movements as quickly and accurately as possible with both hands for 10 seconds. The experimenter demonstrated the motion and provided a detailed explanation before the experiment began. Once the “recording” sign appeared on the screen, participants performed the hand rotation movements. This was repeated 3 times, and there was a 30-second break in between trials. (6) A survey was conducted to collect the demographic characteristics (gender, age, education level, etc). Young participants complete the survey via Google Forms themselves, while for older adult participants, the researcher reads the survey questions aloud, and their responses are recorded in Google Forms by the researcher. After finishing the survey, the participants received the reward.

### Measurements

Based on the video-captured data, we have defined 7 measurements and automatically calculated them by the system (Table 1). The single-hand test indicator assesses wrist flexibility through rotational movements. The time comparison indicator between rotations evaluates changes in hand movement over time, and the angle comparison indicator between rotations assesses changes in hand angle over time. The single-hand test indicators include total rotation count, total rotation time, and total rotation angle. The time comparison indicators between rotations include total rotation time change and the number of rotation angle changes, while the angle comparison indicators between rotations include total rotation angle change and the number of rotation angle changes. Here, the time of rotation change amount represents the total time variation for performing 1 full rotation. Simply put, if the time of rotation change amount is negative, the rotation speed increases as the number of rotations increases; if positive, the rotation speed decreases. The angle of rotation change amount indicates the unit of change in rotation angle. If negative, the rotation angle performed by the participant decreases as the number of rotations increases; if positive, the rotation angle gradually increases [29].

## Results

### User Statistics

This study recruited a total of 70 participants (31 young adults and 39 older adults). In total, 2 young adults were excluded based on the PPT exclusion criteria, resulting in a final sample of 68 participants: 39 (57.35%) in the experimental group and 29 (42.65%) in the control group. The analysis results showed that the average age of the young adults (20-29 years old) was a mean of 22.75 (SD 1.83) years, and the average age of the older adults (65-80 years old) was a mean of 70.36 (SD 3.70) years. Based on the dominant hand, there were 6 left-handed individuals (20.69%) and 23 right-handed individuals (79.31%) among the young adults, while there was 1 (2.78%) left-handed individual and 44 (97.22%) right-handed individuals among the older adults. In terms of educational level, there were 3 (10.34%) highly educated individuals (university graduates or higher) among the young adults and 6 (15.38%) among the older adults (refer to Table 3). This demographic information provides a basis for evaluating the impact of variables such as age, dominant hand, and educational level on hand motion control ability.

**Table 3:** General demographic characteristics of participants (N=68).

		Control group	Experimental group
Age group		Young (20-29 years)	Older adults (65-80 years)
**Gender**			
	Male, n (%)	17 (58.62)	5 (12.82)
	Female, n (%)	12 (41.38)	34 (87.18)
	Age (years), mean (SD)	22.75 (1.83)	70.36 (3.70)
**Education, n (%)**			
	No formal education	—^a^	3 (7.69)
	Elementary school	—	7 (17.95)
	Middle school	—	13 (33.33)
	High school	26 (89.66)	10 (25.64)
	More than college degree	3 (10.34)	6 (15.38)
**Dominant hand**			
	Left hand	6 (20.69)	1 (2.78)
	Right hand	23 (79.31)	44 (97.22)
Purdue Pegboard Test score		13.31	10.67

^a^Not applicable.

### Evaluation Outcomes

A GEE model was used to assess the effects of age group (younger vs older), hand (right vs left), and trials (1, 2, and 3). Table 4 shows the representative statistics for all the measurements that were recorded to give an overview of the results. Among the measures we have defined, only 3 measures (ie, total rotation count, total rotation time, and total rotation angle) have shown a significant effect on the main factors that we have designed. The analysis results are explained for the 3 measurements, including all the interaction effects, are provided in the main text in the following sections.

**Table 4:** Generalized estimating equations analysis techniques (N=68).

Variable and group			Mean		SD		Significance level, *P* value				
							Group		Hand		Group x Hand
**Total rotation count**											
	Young	25.66		7.16		—^a^		—		—	
	Older adults	19.56		7.02		<.01		.24		.85	
**Total rotation time**											
	Young	8.77		1.53		—		—		—	
	Older adults	7.37		1.84		<.01		.18		.46	
**Total rotation time change**											
	Young	–.00		0.09		—		—		—	
	Older adults	–.03		0.14		.32		.33		.16	
**The number of rotation time changes**											
	Young	.11		2.09		—		—		—	
	Older adults	–.18		1.83		.76		.72		.34	
**Total rotation angle**											
	Young	4679.74		3209.04		—		—		—	
	Older adults	2718.68		1235.20		<.01		.61		.17	
**Total rotation angle change**											
	Young	–1.65		45.16		—		—		—	
	Older adults	–2.98		33.22		.75		.34		.07	
**The number of rotation angle changes**											
	Young	.19		3.46		—		—		—	
	Older adults	–.62		3.17		<.58		.50		.48	

^a^Not applicable.

### Total Rotation Count

For the total rotation count, the model revealed a significant main effect of age group. Younger participants performed significantly more full rotations compared to older participants (B=5.29, SE 1.67, *z*=3.16, *P*=.002). Specifically, younger participants completed 5.29 more rotations on average than their older counterparts, suggesting that age significantly influences task performance. A significant main effect of trial was also observed, indicating that performance improved across trials. Participants in trial 2 completed 1.77 more rotations compared to trial 1 (B=1.77, SE 0.59, *z*=3.00, *P*=.003), and participants in trial 3 completed 2.31 more rotations compared to trial 1 (B=2.31, SE 0.72, *z*=3.20, *P*=.001). These findings suggest a learning effect across trials, with participants demonstrating better performance in subsequent trials. The main effect of the hand was not significant (B=–1.00, SE 0.87, *z*=–1.15, *P*=.25), indicating that there was no significant difference between the performance of the right and left hands.

Finally, to explore the significant main effect of trial, post hoc pairwise comparisons were conducted between trial 1, trial 2, and trial 3 using Bonferroni-corrected *P* values to account for multiple comparisons. The comparison between trial 1 and trial 2 revealed a statistically significant difference in performance, with participants showing an improvement from trial 1 to trial 2 (t_67_=–4.87, *P*<.0001, Bonferroni-adjusted *P*=.000021). Similarly, a significant difference was found between trial 1 and trial 3, indicating continued improvement (t_67_=–4.85, *P*<.0001, Bonferroni-adjusted *P*=.000023). However, no significant difference was observed between trial 2 and trial 3 (t_67_=–0.76, *P*=.45, Bonferroni-adjusted *P*>.99), suggesting that performance plateaued between these 2 trials. These results confirm a learning effect between trial 1 and the subsequent trials, with participants significantly improving their performance from the first to the second and third trials. However, no further improvement was observed between the second and third trials.

### Total Rotation Angle

The model revealed a significant main effect of age group, with younger participants exhibiting a significantly greater range of motion compared to older participants (B=1334.37, SE 492.99, *z*=2.71, *P*=.007). On average, younger participants had a 1334-unit larger range of motion than older participants, indicating that age significantly influences performance in this task. There was no significant main effect of hand (B=–202.30, SE 140.47, *z*=–1.44, *P*=.15), indicating no substantial difference in range of motion between the right and left hands. The main effect of the trial showed a trend toward significance for trial 2 compared to trial 1 (B=193.69, SE 101.86, *z*=1.90, *P*=.06), although this effect did not reach conventional levels of statistical significance. However, there was a significant increase in range of motion in trial 3 compared to trial 1 (B=295.09, SE 129.29, *z*=2.28, *P*=.02), suggesting that participants exhibited a larger range of motion in the third trial. For interaction effects, several interaction effects between age group, hand, and trial were also examined. The interaction between age group and hand was marginally different (B=470.23, SE 248.58, *z*=1.89, *P*=.07), suggesting that the effect of hand may differ by age group, though this did not meet the threshold for statistical significance. The interaction between age group and trial was significant for both trial 2 (B=736.10, SE 263.73, *z*=2.79, *P*=.005) and trial 3 (B=644.74, SE 317.01, *z*=2.03, *P*=.042). These results indicate that the younger participants exhibited a significantly larger increase in range of motion across trials compared to older participants, suggesting a learning effect for the younger group as they improved more across trials. Posthoc pairwise comparisons were conducted to further explore the significant main effect of trial, with Bonferroni-corrected *P*-values to account for multiple comparisons. The comparison between trial 1 and trial 2 revealed a statistically significant improvement in performance, with participants showing better results in trial 2 compared to trial 1 (t_67_=–3.68, *P*<.001, Bonferroni-adjusted *P*=.0014, Hedges *g*=–0.19). This indicates a meaningful improvement from trial 1 to trial 2. A significant difference was also found between trial 1 and trial 3, with participants continuing to improve (t_67_=–3.37, *P*=.0013, Bonferroni-adjusted *P*=.0038, Hedges *g*=–0.21). This suggests that participants maintained better performance in trial 3 compared to trial 1.

However, no significant difference was observed between trial 2 and trial 3 (t_67_=–0.41, *P*=.68, Bonferroni-adjusted *P*≥.99, Hedges *g*=–0.01), indicating that performance plateaued between these 2 trials. These results confirm a learning effect between trial 1 and the subsequent trials, where participants significantly improved from trial 1 to both trial 2 and trial 3. However, no further improvement was observed between trial 2 and trial 3.

### Total Rotation Time

The model revealed a significant main effect of age group, indicating that younger participants took significantly longer on the task compared to older participants. Specifically, younger participants had a mean increase of 1.40 seconds in total task time compared to older participants (B=1.40, SE 0.35, *z*=3.94, *P*<.001). This suggests that age significantly affects the time taken to complete the rotations.

There was no significant main effect of hand (B=–0.30, SE 0.23, *z*=–1.33, *P*=.18), indicating no meaningful difference in time between the right and left hands.

The main effect of the trial was marginally different for trial 2 compared to trial 1, with participants taking approximately 0.33 seconds longer in trial 2 (B=0.33, SE 0.19, *z*=1.69, *P*=.09). However, this effect did not reach the conventional level of statistical significance. Similarly, the effect of trial 3 compared to trial 1 was not significant (B=0.32, SE 0.25, *z*=1.28, *P*=.20), suggesting that the overall time taken did not significantly change between the trials.

## Discussion

### Principal Results

Research on digital biomarkers based on behavioral data has primarily focused on gait and hand movement data [10]. Among these, hand movement tasks have gained attention due to their minimal spatial requirements [11,12], absence of fall risk, and the use of low-cost measurement devices. This study investigated whether hand rotation—a task with these advantages—could effectively distinguish age-related differences in motor function.

Accordingly, we hypothesized that there would be significant differences between the young adult group and the older adult group. The results from the GEE analysis demonstrated clear distinctions between younger and older participants across the 3 measures: total rotation count, total rotation angle, and total rotation time. Younger participants consistently outperformed older participants, completing more rotations and exhibiting a greater range of motion, which highlights significant age-related differences in motor efficiency and flexibility. Although younger participants took slightly longer to complete the task, this may reflect a more controlled approach to performing the rotations. Importantly, all 3 measures were effective in distinguishing age groups, proving especially valuable in capturing the declines in motor performance associated with aging. Together, these measures provide a comprehensive profile of motor function, with total rotation count reflecting speed and efficiency, total rotation angle highlighting flexibility, and total rotation time capturing pacing strategies, all of which vary significantly between younger and older adults.

### Detailed Interpretations and Comparison to the Literature

The age-related differences observed in hand rotation metrics likely reflect a combination of cognitive and physical factors. This biomarker, inspired by the link between upper limb motor skills and cognitive function, aligns with prior findings showing that cognitive impairment often accompanies motor declines. Tasks like hand rotation, which require controlled, repetitive movements, depend on cognitive functions such as coordination, spatial awareness, and consistent adjustments—areas that can be affected by age-related cognitive decline. Reduced cognitive capacity could thus contribute to the lower motor performance observed in the older adults group. At the same time, physical fitness factors, including muscle strength and joint flexibility, also decline with age and impact performance on repetitive motor tasks. Although the hand rotation task is low in exertion, it demands a baseline level of physical capability, particularly for maintaining consistent movements across trials. Therefore, this biomarker likely captures influences from both cognitive and physical domains, with declines in either potentially diminishing performance. Future research might benefit from a dual approach, examining both physical fitness and cognitive performance measures in conjunction with this motor biomarker to clarify their relative contributions. This approach could help identify the extent to which cognitive versus physical decline drives the observed performance differences and potentially enhance the biomarker's specificity for early detection of age-related cognitive impairment.

### Implications of the Learning Effect: First Trial as a Practice Trial

The results clearly demonstrated a significant learning effect across trials, particularly for younger participants, who showed substantial improvement from trial 1 to trial 2 and trial 3 in both full rotations and range of motion. The lack of a significant difference between trial 2 and trial 3 suggests that participants reached a performance plateau by the second trial. This indicates that trial 1 likely served as a familiarization or practice phase, during which participants adapted to the task demands. To improve the accuracy of future analyses and minimize the confounding effect of learning, it would be advisable to treat the first trial as a practice trial and focus analysis on trial 2 and trial 3 as representative of stable performance. By doing so, researchers can better isolate genuine performance differences between age groups, unconfounded by initial adaptation or learning.

### Limitations

This study has several limitations. First, the sample size used in the study was limited, which may restrict the generalizability of the results. Future research should increase the sample size to enhance the reliability of the findings. Additionally, this study was cross-sectional, analyzing data at a single point in time. To more accurately understand changes associated with age, longitudinal studies that track changes over time are needed. As the first step in validating digital biomarkers, this study conducted experiments on younger and older adults through hand rotation movements prior to MCI and dementia diagnoses. Although we informed that we were recruiting participants without MCI, we did not conduct an additional screening test. In subsequent phases, we plan to collaborate with Dong-eui University Oriental Medicine Hospital to conduct cognitive tests on older adults diagnosed with MCI and dementia, as well as older adults without cognitive impairment. Through this, we aim to explore and analyze differences in physical, physiological, and gender factors. Furthermore, future research will increase the sample size to study differences between genders and age groups and continue to explore the potential of hand rotation movements as digital biomarkers.

### Conclusions

This study confirmed that hand rotation metrics can effectively distinguish motor performance differences between younger and older adults. The older group showed statistically significant decreases in rotation frequency, angle, and duration compared to the younger group, indicating an age-related decline in motor control abilities. Additionally, a learning effect was observed during repeated trials, suggesting that excluding the first trial may enhance measurement stability in future assessments.

These findings demonstrate the potential of hand rotation as a digital biomarker for capturing age-related changes in motor performance and further suggest its applicability as an early detection tool for MCI and other early cognitive declines. This method can be implemented across various platforms—including webcams, wearable devices, and VR systems—enabling broad applications such as clinical diagnostic support, home-based self-assessment kiosks, and community health screening. In particular, it holds promise as an important assessment tool for the early identification of neurodegenerative diseases and for the development of tailored intervention programs.

Future studies should aim to further refine this hand rotation-based digital biomarker for broader applicability. This study has some limitations, including a relatively small and demographically homogeneous sample, which may limit the generalizability of the findings. Follow-up research involving larger and more diverse populations is essential to improve the generalizability of the findings. Moreover, applying this method to individuals with MCI and other cognitive impairments will help evaluate its effectiveness in detecting early signs of cognitive decline. Expanding the framework to include other motor tasks and integrating multimodal indicators—including cognitive, physical, and emotional factors—could contribute to building a comprehensive and personalized digital health monitoring system for early diagnosis and preventive care.

## Abbreviations

AI: artificial intelligence
GEE: generalized estimating equations
MCI: mild cognitive impairment
PPT: Purdue Pegboard Test
VR: virtual reality

## Data Availability

The dataset generated and analyzed during this study is publicly available on GitHub [30]. The repository contains anonymized hand rotation movement data from younger and older adult participants, along with metadata such as participant group information and measurement variables (eg, rotation count, angle, and time).
